# Embodied Prevention

**DOI:** 10.3389/fpsyg.2022.841393

**Published:** 2022-03-04

**Authors:** Gerd Kempermann

**Affiliations:** German Center for Neurodegenerative Diseases (DZNE) Dresden, CRTD – Center for Regenerative Therapies Dresden, TU Dresden, Dresden, Germany

**Keywords:** reserve, resilience, plasticity, maintenance, dementia, embodiment, walking, exercise

## Abstract

Evidence-based recommendations for lifestyles to promote healthy cognitive aging (exercise, education, non-smoking, balanced diet, etc.) root in reductionistic studies of mostly physical measurable factors with large effect sizes. In contrast, most people consider factors like autonomy, purpose, social participation and engagement, etc. as central to a high quality of life in old age. Evidence for a direct causal impact of these factors on healthy cognitive aging is still limited, albeit not absent. Ultimately, however, individual lifestyle is a complex composite of variables relating to both body and mind as well as to receiving input and generating output. The physical interventions are tied to the more subjective and mind-related aspects of lifestyle and wellbeing in the idea of the “embodied mind,” which states that the mind is shaped by and requires the body. The causality is reciprocal and the process is dynamic, critically requiring movement: the “embodied mind” is a “embodied mind in motion.” Hiking, playing musical instruments, dancing and yoga are examples of body–mind activities that assign depth, purpose, meaning, social embedding, etc. to long-term beneficial physical “activities” and increase quality of life not only as delayed gratification. The present motivational power of embodied activities allows benefiting from the side-effects of late-life resilience. The concept offers an access point for unraveling the mechanistic complexity of lifestyle-based prevention, including their neurobiological foundations.

## Introduction

Aging confronts us with the gradually intensifying experience of how closely body and mind are entangled. What early in life appears a self-evident unity becomes increasingly fragile, when damages and other changes accumulate and the integrity of the mind–body-system is challenged (Kuehn et al., 2018). Dementias, as the extreme, are experienced as a loss of the self. In contrast to the broadly promoted, yet reductionistic strategies for healthy or “successful” cognitive aging (World Health Organization, 2019; Livingston et al., 2020), improved approaches should thus aim at maintaining the “embodied self” in a state that is able to master the challenges of an ever-increasing lifespan. If body and brain change, so does the mind, but their relationship, reflecting the duality of human nature, is far from linear or uniform and not the consequence of simple, accessible causalities.

This insight is not new: the “body and mind problem” continues to be an unresolved challenge to human self-reflection and philosophy. That over millenia the problem has not found a straightforward solution, might also be one reason, why lifestyle interventions that target body and mind separately, fail to unequivocally reach us. While official health recommendations often do imply the existence of a causality from body to mind (“take the stairs and eat your beets” in order to “stay cognitively fit”), this link remains mechanistically vague and the existence of reverse causalities from mind to body is much less emphasized.

This article here explores, also from a neurobiological perspective, some challenges and chances that arise from the fact that body and mind are two undividable aspects of what and who we are, inseparable and not reducible to either of them. Hiking, playing a musical instrument, dancing and Yoga are discussed as exemplary activities and life-style activities that might guide the way for future approaches to healthy and successful cognitive aging that include this perspective. They are examples of complex body–mind activities that emphasize the emergent center that remains untouched by more reductionistic health behaviors. The key to understanding their particular nature and benefit lies in the idea of the “embodied mind.” This concept has an undervalued neurobiological core that would allow to apply the tools of modern neurobiology to the question of how we can achieve and improve prevention.

The idea developed in this article is, however, explicitly not meant as argument against evidence-based recommendations and rigorous standards in scientific studies on identifiable life-style factors [as summarized, e.g., here: Livingston et al. (2020)], which remain necessary and important, but as a complementary perspective that can help to close a conspicuous gap.

It is acknowledged that the present hypothesis article takes a reductionistic approach in that many terms (mind, embodiment, wellbeing, plasticity, etc.) are taken at face validity, although relevant discussions about their exact meaning in this context should be led. The article also assumes a bird’s eye perspective on the psychological, medical and neurobiological literature of lifestyle and lifestyle interventions, including the relevant methodology. For the time being we are not considering many details that ultimately will become relevant. The article is not meant as last word on the proposed concept but rather as an invitation to discuss the idea in an interdisciplinary field. A more in-depth elaboration, including methods, concrete directions of future intervention studies and a more formalized model will have to follow.

## The Embodied Mind in Motion

“Embodiment” in the sense applied here stands for the “*seemingly simple idea that intelligence requires a body*” and that “*not only categorization is grounded in (shaped by) the body, but so is cognition in general, including spatial and social cognition, problem solving and reasoning, and natural language*” (Pfeifer and Bongard, 2006). The concept of embodiment, which originated in both philosophy and cognitive neuroscience has many facets (Meteyard et al., 2012), the key point here being that, irrespective of details in the conceptualization, the brain is not isolated from the body, as the nervous system with both afferences and efferences permeates every bit of it structurally and functionally.

While the “self” is a construct built by the mind, it is inseparable from our bodily existence. The bodily boundaries, densely equipped with sensory receptors, are the relevant interface to the world. Communication with the world happens here, not at the meninges (as the boundaries of the brain) or an ephemeral “soul.” So close is the link to outer experience that virtual reality can, at least on the short term, trick us into illusionary body experiences (Landau et al., 2020).

The body is also the sole reference framework for perception and action that is stable throughout life. Consequently, across our lifespan, we perceive ourselves as living in the same body, even though, objectively, this body changes substantially over the years. But our intuitive self-experience is a unity of the “I” and its body. This is, as pointed out by G.F. Stout, either a fundamental illusion, or must become the basis of all our attempts to the determine the relation of body and mind (Stout, 2012). This is also of relevance when we attempt to increase resilience of brain and mind through bodily interventions, which is the most common idea behind lifestyle-based prevention. But in its unidirectionality this can be clearly only part of the full picture.

For prevention of cognitive decline, we need to target body and mind as entwined, not as separate entities. Again, this is not to say that individual measures, e.g., to promote exercise or balanced diets are not useful, but that by themselves they have limitations that could be overcome with more holistic approaches.

## Limits of Conventional Prevention Concepts

Lifestyle interventions to prevent cognitive decline have proven effectiveness (Kivipelto et al., 2018). It is thus for plausible reasons that the WHO, the Lancet Commission and other institutions base their recommendations on best evidence from epidemiological and intervention studies (World Health Organization, 2019; Livingston et al., 2020). However, this approach, while scientifically and ethically correct, is inevitably not only biased to what could already be studied appropriately but also blind to many interaction effects. In addition, it ignores the large number of additional small factors that sum up to a substantial proportion of the observed or theoretically possible effect, even though they individually fail to reach statistical significance.

Finally, and most importantly, the conventional single factor approach favors easily quantifiable measures that are conceptually straightforward, usually at a physical level, over the complex, supposedly “soft” measures that require the problematic assessment of values, purpose, and social interactions. In contrast, most people value the latter much more highly than the former and intuitively place the plain activities into their larger “softer” contexts. ‘Diet, for example, is not just “nutrition” and the appropriately measured intake of calories, but ideally a broadly fulfilling and highly social activity.’ What comes to mind with “Mediterranean diet” is not just a balanced list of particular healthy ingredients, but an approach to life in a much broader sense, ranging from taste to associations of southern life, that defies the reductionism into which it is squeezed. Consumption of the ingredients as such will to some degree be beneficial as preventive practice (Valls-Pedret et al., 2015), but its acceptance will ultimately depend on the experience of the beneficial connotations that are hard to capture in rigorous scientific studies but whose emotional value might be the most potent driver of health behaviors.

The important non-physical lifestyle factor “education” might at first appear to be an exception of this rule, but in most studies education is not captured as a continuous lifelong activity but as past “educational attainment,” often equal to the highest school degree obtained. The complex dynamic process is reduced to a single number or category. Nevertheless, the early-life academic achievement, independent of any continued education, provides an offset for down-ward sloping cognitive trajectories but does not further flatten the slope of the decline (Nyberg et al., 2021). While this offsetting reserve-like effect is important, the educational foundation that is laid in the past does not necessarily reflect learning-related lifestyles later in life. An interaction effect of continued education with the cognitive trajectories is much more difficult to assess. One attempt has been made in a large study showing that participation in intellectual activities late in life is still associated with a lower risk of incident dementia years later, independent of other lifestyle factors (Lee et al., 2018).

Some attempts have also been undertaken to address the subjectively very highly valued but even more elusive forces such as religious practice, volunteering, optimism, etc. [see Table 1 in Kempermann (2019b)]. Nevertheless, there are some suggestive indications of their positive effects on cognitive aging—not unexpectedly though with by and large weak evidence. Such studies usually rely on self-reports, are burdened with numerous risks of bias and tend to suffer from poor quantifiability. In the end the “immaterial” aspects thus usually remain problematically lifeless and abstract. To overcome this problem, it might be more promising to leave the box closed and assess complex lifestyles as a whole, forfeiting the ambition to isolate relevant ingredients but come to more relevant conclusions about practices that are bundled in real-life anyway.

## The Nature of Life-Style and Life-Style-Based Interventions

Lifestyle-based interventions literally aim at intervening at how people decide to live their lives and how they actually enact that explicit or unconscious decision. They thus require a broad understanding of “lifestyle” itself.

- Lifestyles are stable yet highly personal and individual patterns. How an individual leads his or her life, is expression of personal preferences and shaped by experience. Standard recommendations for healthy lifestyles can only generically address facets of this complexity. Two key scientific questions arise: What are cause and consequence of the inter-individual variability? And what, on the other hand, is the common ground between individuals?
- Personal lifestyles are shaped by gene × environment interactions like all other traits. The genetic component must not be neglected. Environment importantly includes the non-shared factor of individual activity and behavior that is critical for lifestyle interventions to support lasting health. The key scientific question is how this individualizing phenotypic complexity might be captured, modeled and analyzed.
- Lifestyles are about wellbeing in the presence. What we accept as “good for us” is only to a small part governed by rational decisions and the expectance of delayed gratification. Lifestyles are also subjective with respect to the quality of the experience and the assigned emotional value. The question is how individual wellbeing as a state of balance is a consequence of lifestyle (and its conscious changes).

## Physical Activity as a Common Denominator

Given the obvious complexity it has always puzzled people that among the identifiable components of lifestyle, plain physical activity appears to play a dominating role (Norton et al., 2014). Ultimately this might not be so surprising, however, as nervous systems have evolved to allow active mobility and as all output of the brain is ultimately motoric. Physical activity immediately engages the brain on both the input and output side. Physical activity is a motoric output of the brain that generates sensory input, both from the body itself and environment. The intrinsic feedback happens directly through efference copies and indirectly measuring the effects in the periphery.

Proprioception and balance provide constant and massive input from the moving body to the brain. In addition, rhythms of neural activity resonate with patterns of physical movement, such as during walking, and promote theta rhythms in the hippocampus, which facilitate memory consolidation (Terrazas et al., 2005).

It is therefore plausible that all higher brain function is in one way or another linked to mobility and that interaction with the world and movement are inseparable. The world needs to be physically approached and probed for cognition and affective behavior to happen. Action always precedes cognition. Embodiment is thereby not a static *a priori* but a continuous process, which plastically shapes the mind through navigation of the world. In that sense, physical activity is a proxy for body and mind in motion and exercise is effective as lifestyle factor not only because of non-specific invigorating systemic effects but also because it re-establishes a natural, fundamental dynamic connection between body, brain and mind.

Problems arise, if this relationship is lastingly severed. In the context of preventive practice for healthy cognitive aging, the idea is that cognition suffers, the reserve formation shrinks and resilience is reduced, if the brain is deprived from opportunities for active engagement through physical interaction and the resulting sensory feedback from the body and the exterior senses. Conversely, therefore, engaging in physical activity is probably the single most valuable preventive action that anybody can take to re-establish that body–mind connection. But as the brain is required to initiate and maintain physical activity in the first place, also the associated affective states and the resulting sense of a “quality of life” are essential. They drive and motivate the activity and allow to evaluate and value it. The triad of emotions, cognition and motor activity is the key to behavior. The embodied mind is essentially an “embodied mind in motion” and this idea could become the nucleus to a general neurobiological concept of resilience and prevention.

## The Real-Life Complexity of Life-Style and Life-Style Based Interventions

But focusing on motoric activity, even though it is extremely effective, is not enough, also because people differ substantially in how easily they engage in it and maintain a lifestyle with regular exercise.

Systematic categorization of the large number of potentially preventive practices, for which at least a certain level of scientific evidence exists, revealed that they appear in fact partly antagonistic and create suggestive tension fields (Kempermann, 2019b). They also add dimensions that are not covered by the well-supported interventions like physical activity. In sum, these potential factors as a whole, more than the well-researched “usual suspects” (exercise, non-smoking, balanced diet, etc.), affect both body and mind and they reflect receiving input as well as generating output. Together are reflecting the embodied mind in its active interaction with the world.

Given that—outside experimental situations—many of these describable subfactors will act in concert, the focus on large effect sizes only might be thus misleading. Both the highly polygenic inheritance and complex individual environmental trajectories (shared and non-shared) will influence the overall outcome. Lifestyle is ultimately a non-linear, non-additive concept and the most successful preventive lifestyle profiles will be highly polyfactorial and multimodal. Presumably they must keep some balance along the seemingly orthogonal trajectories of the matrix. They are intrinsically interactive, which is also reflected in the very large communalities that the known factors (especially physical activity) exhibit in the large epidemiological studies (Norton et al., 2014). For most of the activities, however, such overlap and shared influence has not yet been calculated. It thus seems that we need much more complete models of lifestyle. And at the same time we must learn to understand the relationship between the key dimensions of lifestyle. The resulting reductionism would be considerably different from the one practiced now.

## Wellbeing as Center of Life-Style Interventions

Complex organisms achieve and maintain their bodily equilibria through autonomous systems as well as through willful actions. Emotions signal the diversion from the equilibria and initiate activities. Long-term quality of life and wellbeing are ciphers for the experience of homeostatic states. The emotional connotations are arguably stronger drivers of lifestyle than reason. Unhealthy behaviors result from conflicting emotions and motivations in such situations.

Incidentally, the perceived value of a balance between physical factors on one side and mental, spiritual or social factors on the other and between acts of receiving and of giving is also central and essential to Western and Eastern wisdom traditions on how to lead a good life. These are centered around strong emotional cores.

Invariably these traditions tend to particularly emphasize the spiritual domain but they often involve physical practices and often nutritional guidance as well. Importantly, however, they offer more than regimes of activities. They address the entire human being and they all have an immaterial, spiritual center, transcending the physical world. While they are thus often perceived as in conflict with a scientific world view, they in fact identify central questions of the human condition and are immediately relevant to the lives of millions. Given their presence and influence, it is hard to argue, why they should not be the subject of scientific investigation in a context, which so clearly calls for their inclusion. Such perception might also change, as, for example, the neuroscience of mindfulness and meditation, previously prime examples of such elusive, soft activities has become generally accepted and has led to tangible results.

At the same time, however, many clinical studies in other contexts (especially in oncology) use quality of life measures, assessed by standardized scales and questionnaires as end-point (Soni and Cella, 2002), recognizing that where the patients stand emotionally and how they feel, might often be more relevant than objective clinical endpoints, biomarkers, scores, signs and symptoms alone. In fact, a “benefit” visible in physical parameters or biomarkers but nevertheless associated with prolonged suffering is widely rejected as irrelevant.

Consequently, palliative therapy for fatal disease at the end of life orients itself almost solely on quality of life measures. It has been argued that for old and oldest age, also in the absence of palliative conditions, quality of life should be the decisive variable to guide all medical decisions. It thus is worth further discussion, whether prospectively, in a preventive context and at much younger age, which under the current demographic trends will likely lead to very old age and a great likelihood of multimorbidity—this same variable should not receive even more appreciation and attention.

Quality of life and wellbeing certainly cannot be the sole target of holistic interventions to promote a beneficial, healthy lifestyle, as it is obvious that on a short-term many irrational, unhealthy and potentially damaging behaviors and habits might produce an instantaneous increase in quality of life. But these short-term hedonistic qualities must always be seen in their life-course context. There are in fact different relevant forms of wellbeing coexisting within the same life. This idea again alludes to the religious and wisdom traditions, which tend to distinguish “worldly” short-term bliss from a greater good. That insight, however, is neither religious *per se*, nor does it necessarily comprise elements of transcendence. But religion is one possible answer to this central question and not surprisingly, thus, religious beliefs and practice have also been found to be associated with health benefits and successful cognitive aging (Peteet and Balboni, 2013; Hosseini et al., 2019; Khalsa and Newberg, 2021). Wellbeing as opposed to momentary bliss is usually associated with states of balance. Healthy lifestyles must achieve such balance in order to become sustainable. Scientifically, this means that understanding the nature, origins and mechanisms of homeostatic states are central to understanding, how we can lead our lives in order to remain prepared for oldest age.

A Research Topic in Frontiers in Aging Neuroscience explicitly assembled reports on studies on “Interventions to Promote Wellbeing in Old Age,” presenting numerous interventions, from physical (the large majority) to cognitive and spiritual. A comprehensive editorial outlined the conceptual framework spanned out by this range of interventions. What remained less clear, however, is how all (or many) of the activities might actually integrate to manifest as wellbeing and how wellbeing in turn might act back upon endpoints like cognition, general fitness, etc. (Foster et al., 2019).

## Beneficial Lifestyles Have Societal Not Only Individual Dimensions

Beneficial interventions that, on the contrary, decrease subjective quality of life face a difficult uphill battle. Health behaviors that are solely relying on reason but acutely evoke negative emotions have poor chances of becoming stable. This applies to exercise regimens, diet recommendations, educational objectives and others. Many standard recommendations for healthy cognitive aging rely on the unrealistic prerequisite that they must be embraced emotionally and become naturally integrated into the quality of life. How this is going to happen is usually left to chance and depends on genetic predispositions, personality traits, past experience and circumstances.

On the other hand, some promising developments might guide the way. The continuing decreases in the risk to develop dementia in consecutive age cohorts (roughly 16% per decade), as detected in the Framingham study, the Rotterdam study and others, have been credited to environmental and lifestyle changes over the past decades that were part of general turns in societal perception and often reflected overarching political decisions (e.g., regarding pollution, smoking ban, etc.; Wolters et al., 2020). They were not, however, the consequences of collective decisions to adopt beneficial lifestyles according to evidence-based check lists with delayed gratification. Socially and environmentally induced behavioral changes can become common sense and greatly matter.

Studies on centenarians and the so-called “blue zones”—regions of the world with an unusually high number of healthy very old people—also revealed that, besides the contribution of the local gene pool, the positive effects on lifespan and health in old and oldest age were usually not due to intentional individual changes in lifestyle but associated with an established way of life, deeply rooted in the society and a shared sets of values. While many attempts have been made to mechanistically deconstruct the blue zones and the lifestyle trajectories of centenarians (again besides deciphering potential genetic bases), the studies rather unanimously point to the existence of a pre-existing mindset that is not focused to achieving old age or health but profoundly anchored in the presence. The subjectively high quality of life reported by many of these oldest-old people is also associated with often rather modest economic means and a high resilience to adverse life events, including war. For them, becoming old in cognitive health is the often (but not even always) welcome side effect of a life at peace with themselves. While it is scientifically challenging to capture this aspect and enter its measure into a comprehensive model of resilience, the reports support the notion that variables other than the objective physical parameters matter and that populations that (besides genetic predispositions) have achieved collective life styles that support healthy cognitive aging and longevity do so implicitly and without perceived hardship on a path toward a distant goal.

Together these insights indicate that, besides providing supportive legal and political frameworks, the role of society in forming and sustaining lifestyle goes much deeper.

While it will not be realistic to actively emulate the complexity of blue zones and to call for societal change as a feasible first step, the most important aspect to learn from these environments is the holistic nature of the life-style they represent, centering around a subjective quality of life and peace of mind. The problem, of course, is that exactly these variables are latent and evade reductionistic experimental approaches. But as little as polygenic effects can be pieced together bottom-up from series of pseudo-mendelian experiments on individual genes, lifestyle is much more than the sum of quantifiable measures but has emergent qualities.

A reasonable first step toward comprehensive preventive measures that relate to lifestyle in a deeper sense and to the embodied mind in motion is therefore to explore known activities that are partly reductionistic in that they are still identifiable “interventions,” rather than as complex as life itself, but are experienced as holistic and increasing wellbeing now and not only benefits later.

## Examples of Body–Mind Life-Style Activities Lead the Way

### Hiking

Hiking is the combination of physical activity with experience of nature, either in deliberate solitude or as social activity. Intensity levels are low to medium at most times, but endurance can be a major factor, when hikes become long and the terrain challenging. Important physiological characteristic of hiking is the regular pace and sensory richness, including proprioceptive input on varying undergrounds. A meta-analysis of 26 studies on long-distance hiking revealed a positive association with mental health, most notably stress reduction, but less clear for general wellbeing (Mau et al., 2021).

Given the popular attention that hiking receives as potential health behavior, the number of actual studies is, however, surprisingly low. That hiking exposes to an experience of nature is considered a central element [see the extensive narrative review by Mitten (2009)]. Another aspect that is considered essential is the room the that long-distance hiking provides for personal reflection (Mau et al., 2021).

Long-distance hikers were studied in a questionnaire study (Mayer and Lukács, 2021). The authors summarize the findings as: “Hikers had mainly intrinsic motivations to complete long distances including overcoming new challenges, finding the physical boundaries, experiencing a state outside the comfort zone, belonging to a special group with similar interest and attracted by the beauty of nature. Overcoming all these embodied in a flow experience that took them further to perform the new long-distance trails.”

There is an overlap between the experience and effect of hiking with long-distance running, but for runners, the sportive aspect usually dominates. Nevertheless, many long-distance runners also emphasize the meditative qualities of long runs (“flow”) and for many running in the outdoors stands for a primal interaction between the body and nature. Flow is characterized as state of enjoyment and reduced self-awareness that occurs during an optimal task performance with low demands to attention. There are still relatively few studies on the physiology of the “flow” state but it appears to be a central moment in explaining the perceived or real neural effects of these activities, deserving further attention.

An impressive 2021 retrospective study on 200,000 participants of Sweden’s long-distance cross-country ski race Vasalopet, who were followed for up to 21 years, revealed a strong association of physical activity with a reduced risk to develop anxiety. Participation in the popular race has been taken as indication of general physical activity, as the race requires extensive preparation. But cross-country skiing is also a classical outdoor activity, and people in the North engage in it especially during the dark period of the year. The authors write: “We identify a need for future studies to gain deeper knowledge about the impact of these confounding psychological factors, taking both environmental, genetic, and epigenetic background into account.” What appears to be a confound from the perspective of studying abstract physical fitness, might actually represent a very interesting contributing factor by itself (Svensson et al., 2021).

The assumed role of the “green outdoors” on the brain has been addressed in a study with unprecedented depth. In longitudinal series of multiple MRT scans over 6–8 months, Kühn et al. (2021) found that the time spent outdoors was positively associated with gray matter volumes in the right prefrontal cortex and self-reported positive affect (after controlling for numerous parameters, including “hours of sunshine”).

Hiking (or walking) appears as the least complex example of a body–mind activity with beneficial effects on the brain including increasing resilience (Tomata et al., 2019).

### Playing a Musical Instrument

Playing the musical instrument might be the best-known and best-studied holistic intervention to date. A relatively strong case can be made for a wide range of positive effects of music on the brain. Playing a musical instrument is an activity that combines various aspects of activity, from motoric to cognitive, often including social interaction. The position of music in the evolutionary perspective is enigmatic. Music, being clearly an achievement of civilization must root on primordial functions and structures that evolved with the brain. This implies that besides all positive aspects of music itself, music is always also a representation of a more general principle. The embodied mind in motion might be a coarse yet fitting description of this condition. Unlike in the example of hiking, musicians are exposed to the experience of a world of its own, produced by the mind, alone or with others, and related to a skillful motor activity in the world. Music is at the same time a language directly speaking to the emotional brain and to the intellect and has a strong social component. There is also anecdotal evidence of particular longevity among (classical) musicians, especially conductors.

Again, relative to the public attention that playing an instrument attracts as a putative approach to prevention of dementia, the number of actual studies is slim. A meta-analysis over three studies of high quality described an impressive 59% reduction in the risk of developing dementia, but cautioned that the size of the evidence base is limited, the studies cover low numbers of participants and causality cannot be established (Walsh et al., 2021). Self-reported musical activity has also notable socio-economic covariables. Nevertheless, a cohort study which assessed the effect of the frequency of playing music in mid-life on later-life cognition revealed that the most active musicians had 80% greater odds of being in the top cognitive decile (Walsh et al., 2021).

Singing will be the most essential form of “playing an instrument,” requiring nothing but the body and breath as means to physically produce music. The relationship between body and mind is rather obvious here. Singing in a choir, which also involves social aspects, consequently has expected effects on quality of life (Johnson et al., 2013). Another study found no effects on cognition (Pentikäinen et al., 2021). Reported subjective benefits, however, were extremely positive in the largest survey to date (Moss et al., 2018), suggesting that the individual interaction effect of objective and subjective variables needs to receive more attention.

### Dancing

Regarding the beneficial effects of dancing on successful cognitive aging, reference is usually made to the impactful report by Verghese et al. (2003), which was a prospective observational study of self-reported leisure activities. To date, there is an overall relatively large literature on the positive effects of dancing on cognition. Meta-analyses of the available literature support positive effects of dance interventions, for example on global measures of cognition, on executive functions and on memory performance (Meng et al., 2020).

When Karkou and Meekums (2017), however, attempted a systematic review for the Cochrane data base of dance therapy for dementia, they did not find studies that could be included into their analysis, because none was conducted by a certified dance movement therapy practitioner. This criterion might be counterproductive for a general evaluation of dance as effective intervention and contradicts the idea of employing activities that people can pursue on their own and integrate into their daily lives. A less restrictive review of essentially the same literature provided support of the idea that dancing therapy would have positive efficacy for cognitive, physical, emotional and social performance in dementia, but also cautioned that the overall quality and rigor of the studies was questionable (Klimova et al., 2017). A case study with a streamed dance course delivered to participants in care homes concretely addressed the aspects of embodiment and highlighted effects on creativity and social inclusion rather than classical endpoints such as “cognitive performance” (Kontos et al., 2021).

This range of statements showcases the problematic evidence situation for dancing as lifestyle intervention for therapy or secondary prevention. It also underscores that the vast majority of available studies does not prominently take on a body–mind perspective, appreciating dancing as a holistic, embodied activity with preventive (side-) effects but rather as a conventional physical intervention.

Accordingly, the frequently found statements that more research would be needed to address the question, whether dancing can lead to more cognitive benefits than other types of physical activity and exercise (Hewston et al., 2021), is understandable from a reductionistic point of view but misses the central question of wellbeing as driver and result as well as a holistic approach to the highly multi-modal and complex intervention “dance.”

In the reported studies, dancing usually is ballroom dancing, folk dancing or explicitly therapeutic forms of dance, but rarely the more professional versions of ballet and modern dance (Karpati et al., 2015).

### Yoga

Yoga differs from the other mentioned activities in that it explicitly addresses both body and mind. Its primary objective is related to the self of the person engaging with the activity, not to nature, music, or social interactions, etc., rendering it more introspective. For many, Yoga and similar activities are attractive because of their immediate effects on bodily and spiritual wellbeing, not because of a delayed health effect or through competition, as in other sports. There are diverse Yoga styles, of which the most relevant in the context or prevention and resilience probably are combinations of dynamically flowing series of poses with elements of meditation, linked by a focused awareness of breathing. Because of the great number of styles and lacking standards for research, the body of scientific literature on Yoga, while being vast, remains inconclusive. Nevertheless, it is the only activity, for which the body–mind aspect has been explicit subject of the research, and as problematic as the overall level of evidence still might be, there is also no doubt that practicing Yoga (as well Tai Chi, etc.) can have numerous positive effects on cognition, also in older adults (Wu et al., 2019). The growing interest in Yoga and its health effects, however, is currently leading to an increasing number of studies with greater quality. It remains to be hoped that on the longer run they do not attempt to view the holistic intervention through the lens of the classical reductionistic single-intervention approaches but develop novel formats that also incorporate embodiment as an essential neurobiological principle and embrace the complexity of the intervention.

As there is already an increasingly solid evidence on structural and functional effects of Yoga on the brain (Gothe et al., 2019), including some first results on cellular aging (Tolahunase et al., 2017).

The point is not, whether any component of Yoga might by itself and stripped from the other components might already have similar effects as Yoga as a whole, but that Yoga has emerging benefits that surpass the addition of identifiable components. Improved wellbeing now and in the future plus health benefits will be more important than health benefits alone, even if the latter were larger in a different context. Wellbeing matters.

## The Neurobiology of Embodied Prevention

**Figure 1:**
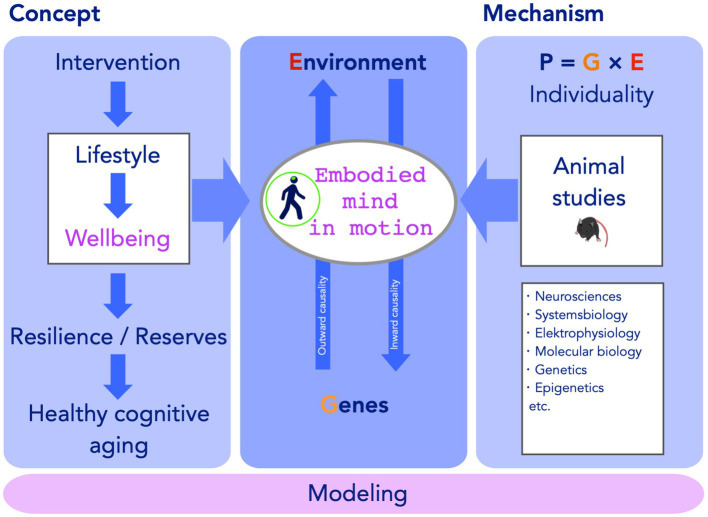
Embodied prevention. The concept underlying lifestyle interventions for healthy cognitive aging is based on the assumption that a lifestyle-changing intervention results in an increase in resilience in old age and a preservation of reserves. The emphasis here lies in the fact that in order to be sustainable, lifestyle must also have motivating effects on wellbeing now. The embodied mind lies at the center of this context, because body and mind cannot be thought separately in this context. Behavior in the environment and the underlying genetic predispositions and causes are linked in a bi-directionality chain or network of causalities, intersecting in the embodied mind in motion. Animal studies and other types of reductionist research can aid understanding this core concept and unraveling mechanisms underlying the equilibria of wellbeing, the embodiment in motion itself, and the interindividual variation of this effect, by building upon the fundamental principle that any phenotype and its variation draws from an interaction of genetic and non-genetic causes. Here the non-shared factor of the environmental effect, which includes individual behavior and actions is of particular interest. Future models of lifestyle based embodied prevention need to include all three pillars.

For a deeper understanding of complex life-style “interventions” that address body and mind, two complementary approaches must be taken. The first is to improve studies in humans engaging in the actual activity, the second to better understand the underlying fundamental biological principles. These must be seen together and the resulting research must be highly interdisciplinary in scope (Figure 1).

The examples of hiking, yoga, etc. show that such studies are very challenging, if the analysis should go beyond the reductionistic approach as taken for identifiable single factors and reach the emergent qualities of the intervention. The additive method of conventional multi-modal trials [such as FINGERS (Ngandu et al., 2015)] is ultimately not sufficient, because the holistic interventions cannot be easily deconstructed into factors without damaging the critical interaction effects and by ignoring present wellbeing as driving force of success. Mechanistically, the bottom-up approach is bound to fail in much the same way as it is impossible to understand complex polygenic traits from the analysis of single associated genes (Boyle et al., 2017). A first meta-analysis of multidomain interventions did not yield evidence of clear benefits (Hafdi et al., 2021).

Whereas the existing large-scale trials still support the notion of an additive and complementary effect in multimodal interventions, most concrete prospective intervention studies on hiking, dancing, yoga, etc. have as yet yielded limited evidence on the applied objective scales, even though, qualitatively, participants described substantial subjective benefits. These highlight the limits of standardization and underscore the individuality (and subjectivity) of the response in life-style interventions. The impact of this subjectivity gap for basic research has as yet received little attention. This is no wonder, given that many essential concepts like subjectivity and wellbeing cannot readily be approached and measured in reductionistic settings. The same applies to the activities themselves: hiking, playing a musical instrument, dance and yoga cannot be studied in laboratory animals.

While this is true, basic neurobiological research, especially with a systems biology perspective, can nevertheless critically help addressing these questions, because the biological perspective zooms in on the evolutionarily conserved fundamental mechanisms that underlie the adaptability to the challenges of long life in a dynamic complex world. The depth of phenotyping under controlled conditions, the possibility to control or manipulate genetics and the environment, and longitudinal tracking and monitoring offers unique opportunity to study the embodied mind in motion under reductionistic conditions, even though the elements of subjectivity and quality of life largely have to be left out. Embodiment is, after all, a profoundly biological concept, even though it was developed in other contexts. In addition, embodiment is part of exciting new concepts in neurorehabilitation based on virtual reality to target declining spatial memory and navigation (Tuena et al., 2021).

In basic neurobiological research, the vast animal literature on “enriched environments” is concerned with the concept that exposure to environments that induce behavioral activity and learning, fundamentally shape the brain, its connectivity and its functions (van Praag et al., 2000; Nithianantharajah and Hannan, 2006; Kempermann, 2019a). Although the paradigm has not yet been explicitly brought into connection with the idea of embodiment, it actually captures the processual aspect of embodiment by highlighting the dynamic link between form and function, called plasticity. Behavioral activity matters for brain integrity and function and this interaction is reciprocal.

A neurobiological perspective on “lifestyle” introduces a new and different reductionism to the study of lifestyle. This reductionism embraces data-driven and systems biology approaches to take head-on the complexity of holistic lifestyle interventions and activity-based resilience by using, for example, model organisms.

Any phenotype—and successful cognitive aging is an observable trait and thus biologically speaking a phenotype—is determined by the interaction of genetic and environmental factors. This interaction can be explored in animal studies by targeted manipulations that are impossible in humans.

Both the risk of and the resilience against neurodegenerative disease and impaired cognitive aging are complex quantitative traits with massively polygenic inheritance but also a strong non-genetic component—hence the impact of lifestyle. What we perceive as specifically human components, e.g., subjective experience and wellbeing, are to a large extent part of the environmental factor. The nature of the interaction effects, however, can be explored with surrogate parameters, assumed homologs of “life-style” in animals.

The term “environment” is a complex construct that goes beyond the physical outer environment. That component, the non-shared environment, includes the ways we interact with the world, transform and shape it, our social interactions, all behaviors of the individual and of others, and many other identifiable aspects that together build the variable individual response to a challenge.

In a highly reductionistic study in mice, it was possible to isolate the impact of the so-called “non-shared environment,” that is the component of the environmental factor to phenotypic variation that consists of the individual behavior even in an environment that is shared and—which is possible in these studies—if the genetic background is kept constant (Freund et al., 2013). That study revealed that individual activity in fact matters, even though in naturalistic settings and especially in humans, the situation must inevitably be more complex and the effect sizes will vary. The effects are also dependent on the individually variable impact of the genetic and the shared non-genetic component on the activity. Irrespective of this, some key concepts of that study (the relationship between exploratory behavior or territorial coverage and brain plasticity) has already been translated to a human study (Heller et al., 2020).

The inward (from behavior to genes) and outward (from genes to behavior) pointing chains or networks of causalities are interdependent (Kempermann, 2019a). The level of cells, tissues and systems is a tangible place where the effect of the reciprocal interaction of these intersecting chains of causality are concentrated and thus can be studied. This, together with the superior role of motor functions (as discussed above), establishes a neurobiological construct of embodiment that emphasizes dynamical change.

In addition, the idea of embodiment has more recently also been explicitly extended to include cellular processes, including “cellular memory,” immunity, the microbiome, etc., arguing that aspects of what we used to consider brain functions are in fact distributed to the body and thus inseparable from it (Verny, 2021).

Ultimately, below all this lie evolutionary questions. Like anything else in biology, also lifestyle-based resilience and the hypothesis of the “embodied mind in motion” will make sense only in the light of evolution. Lifestyle is not only a social construct and a fashionable label for individual actions, but also a possibly ill-chosen term for a fundamental principle of life that deserves neurobiological attention.

## Conclusion

The proposed framework of the “embodied mind in motion” intends to improve the limitations of a common perspective on the life-style based promotion of healthy cognitive aging by shifting the focus from external (single interventions with large effect size) to internal (wellbeing as mediating endophenotype). By emphasizing communality between known factors, their partly antagonistic nature and the potential impact of a large number of factors with small effect size the concept allows for accommodating large inter-individual variability and the underlying real-life complexity in preconditions, personal biographies, and varying circumstances. By rooting in an evolutionarily conserved key relationship between movement and cognition the increase in complexity (by moving from few factors to all factors) becomes approachable in reductionistic experiments, including in animal studies.

## Data Availability Statement

The original contributions presented in the study are included in the article/supplementary material, further inquiries can be directed to the corresponding author.

## Author Contributions

The author confirms being the sole contributor of this work and has approved it for publication.

## Conflict of Interest

The author declares that the research was conducted in the absence of any commercial or financial relationships that could be construed as a potential conflict of interest.

## Publisher’s Note

All claims expressed in this article are solely those of the authors and do not necessarily represent those of their affiliated organizations, or those of the publisher, the editors and the reviewers. Any product that may be evaluated in this article, or claim that may be made by its manufacturer, is not guaranteed or endorsed by the publisher.
